# Cofilin 1 induces the epithelial-mesenchymal transition of gastric cancer cells by promoting cytoskeletal rearrangement

**DOI:** 10.18632/oncotarget.16608

**Published:** 2017-03-27

**Authors:** Haibo Wang, Lide Tao, Feng Jin, Hao Gu, Xiaojun Dai, Tengyang Ni, Jun Feng, Yanbing Ding, Weiming Xiao, Yayun Qian, Yanqing Liu

**Affiliations:** ^1^ The Affiliated Hospital of Yangzhou University, Yangzhou University, Yangzhou 225000, China; ^2^ Clinical Medicine College of Yangzhou University, Yangzhou 225000, China; ^3^ The State Administration of Traditional Chinese Medicine Key Laboratory of Toxic Pathogens-Based Therapeutic Approaches to Gastric Cancer, Yangzhou 225000, China; ^4^ Institution of Combining Chinese Traditional and Western Medicine, Medical College, Yangzhou University, Yangzhou 225000, China

**Keywords:** CFL1, gastric cancer (GC), cytoskeleton rearrangement, epithelial-mesenchymal transition (EMT)

## Abstract

Epithelial-mesenchymal transition (EMT) is an important biological process whereby malignant tumor cells obtain the ability to migrate, invade, resist apoptosis and degrade the extracellular matrix. We found that Cofilin1 (CFL1) expression was elevated in clinical gastric cancer specimens and correlated with biomarkers of EMT in BGC-823 gastric cancer cells. BGC-823 cells exhibited EMT phenotypes and increased metastatic ability when induced by TGF-β1. By contrast, BGC-823 cells transfected with Lv-siRNA-CFL1 did not exhibit EMT phenotypes under the same inducing conditions. As CFL1 expression increased, EMT cell filopodia stretched out. In addition, the ultrastructures observed using transmission electron microscopy indicated that silencing of CFL1 markedly inhibited depolymerization of fibrous actin and cytoskeletal reorganization during EMT. Similar results were obtained *in vivo*. These findings demonstrate that CFL1 induces EMT by promoting cytoskeletal rearrangement. Our results may provide the basis for developing new anticancer drugs to inhibit CFL1.

## INTRODUCTION

According to cancer statistics from 2015, gastric cancer (GC) was the cancer with the highest incidence and mortality in China [1]. Although GC patients can benefit from early diagnosis and standardized surgical treatment, their overall survival is far from satisfactory [2]. Most tumor invasion and metastasis has already occurred by the time patients see a doctor [3]. The invasion and metastasis of GC are important factors preventing improvements in the patient survival rate; therefore, inhibiting these processes is a critical treatment strategy [4].

The epithelial-mesenchymal transition (EMT) is the biological process whereby malignant tumor cells obtain the ability to migrate, invade [5, 6], resist apoptosis and degrade the extracellular matrix [7, 8]. Cofilin 1 (CFL1) is an important regulatory factor of cytoskeletal reorganization in tumor cells, and its expression strongly correlates with the EMT of tumors [9–11]. CFL1 may well become a new target for the treatment of malignant tumor cells undergoing the EMT, invasion and metastasis [12–15]. However, the specific function of CFL1 in the EMT of GC cells remains unclear [16].

In the present study, the expression of CFL1 and its relationship to the EMT was analyzed in clinical GC samples and GC cells. In addition, the mechanism whereby CFL1 is involved in the EMT in GC was assessed both *in vitro* and *in vivo*, with a particular focus on cytoskeletal reorganization.

## RESULTS

### Expression of CFL1 in GC

**Figure 1:**
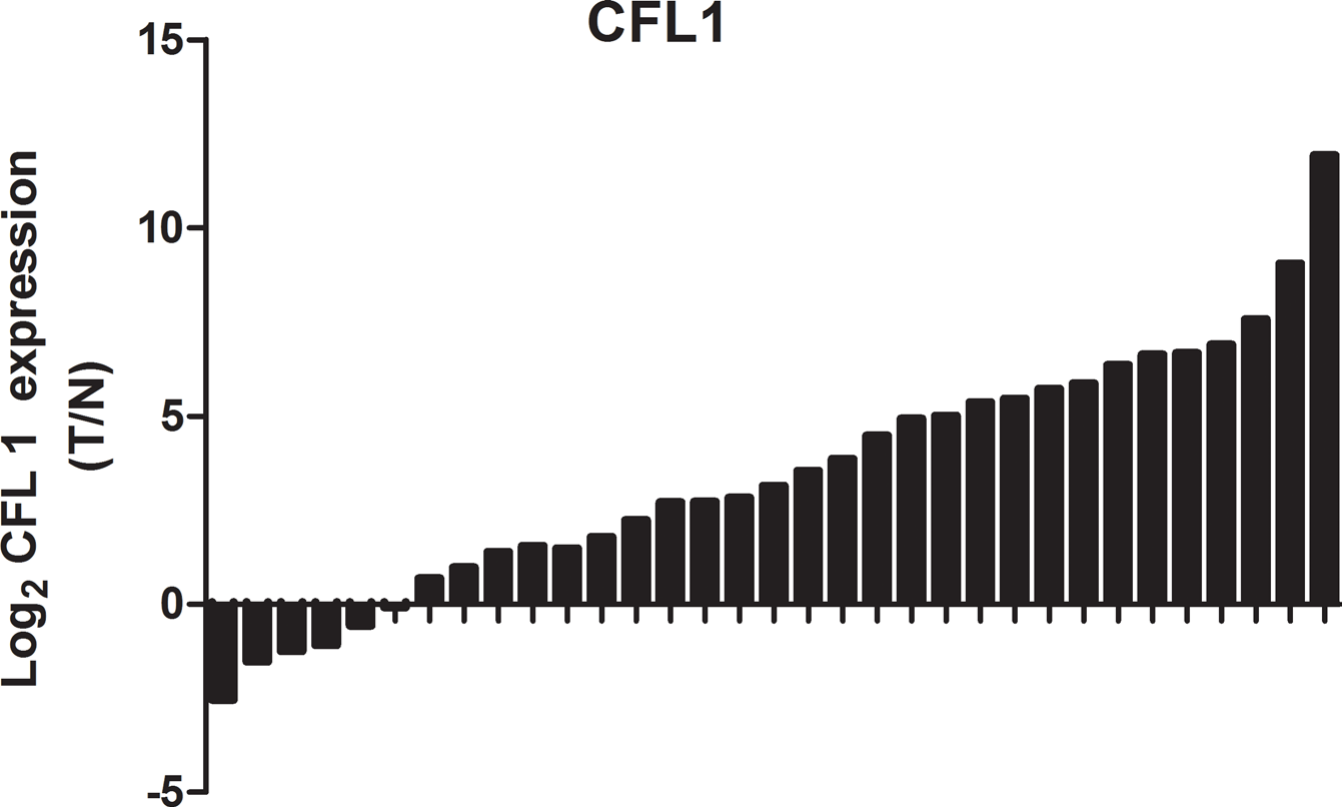
The expression of CFL1 was markedly higher in tumor tissues than in matched adjacent normal tissues

To determine the expression of CFL1 in GC tissues, we performed western blotting on 33 pair clinical samples and compared the results with those of the corresponding normal gastric tissues. CFL1 expression was markedly greater in tumor tissues than in control normal stomach tissues (Figure 1). To further confirm these results, we also quantified the expression of CFL1 in 33 pairs of frozen GC specimens. Consistent with the results obtained above, the expression of CFL1 was markedly higher in tumor tissues than in matched adjacent normal tissues. These results indicated that CFL1 was consistently upregulated in GC.

### EMT model and changes of cell morphology

**Figure 2:**
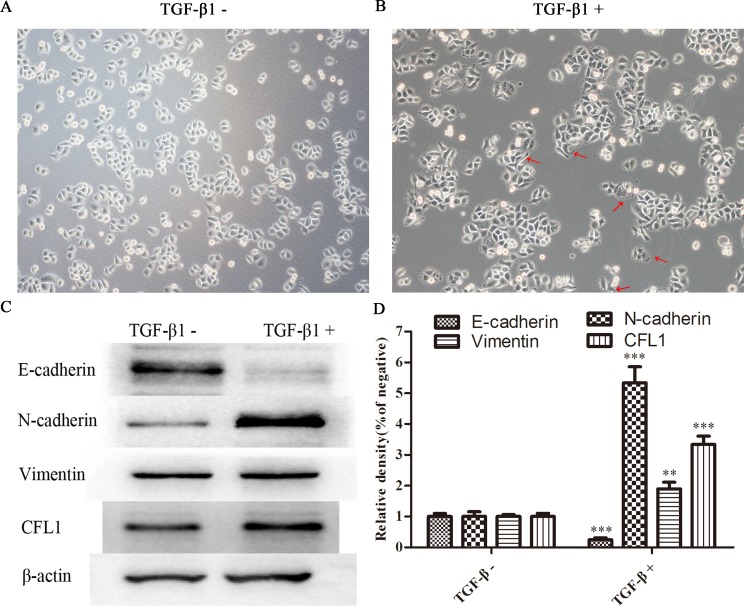
**(A** and **B)** TGF-β1 successfully induced the EMT in BGC-823 cells. **(C** and **D)** EMT biomarker expression was determined. E-cadherin expression was obviously reduced in TGF-β1-treated cells, while N–cadherin, Vimentin and CFL1 expression were markedly enhanced(100×), ** p<0.01, ***p<0.001.

To study the involvement of CFL1 in the EMT, we used TGF-β1 to induce the EMT in BGC-823 cells. The BGC-823 cell morphology was greatly altered after the cells were cultured with 10 ng/mL TGF-β1: the quadrilateral morphology became more spindle shaped. In addition, rich microfilaments endowed cells with a strong ability to migrate and invade (Figure 2A and 2B). In Western blotting of EMT biomarkers, E-cadherin expression was obviously reduced, while N-cadherin, Vimentin and CFL1 expression were markedly enhanced in TGF-β1-treated cells (Figure 2C and 2D). Thus, TGF-β1 successfully induced the EMT in BGC-823 cells.

### The correlation between CFL1 expression and the EMT

**Figure 3:**
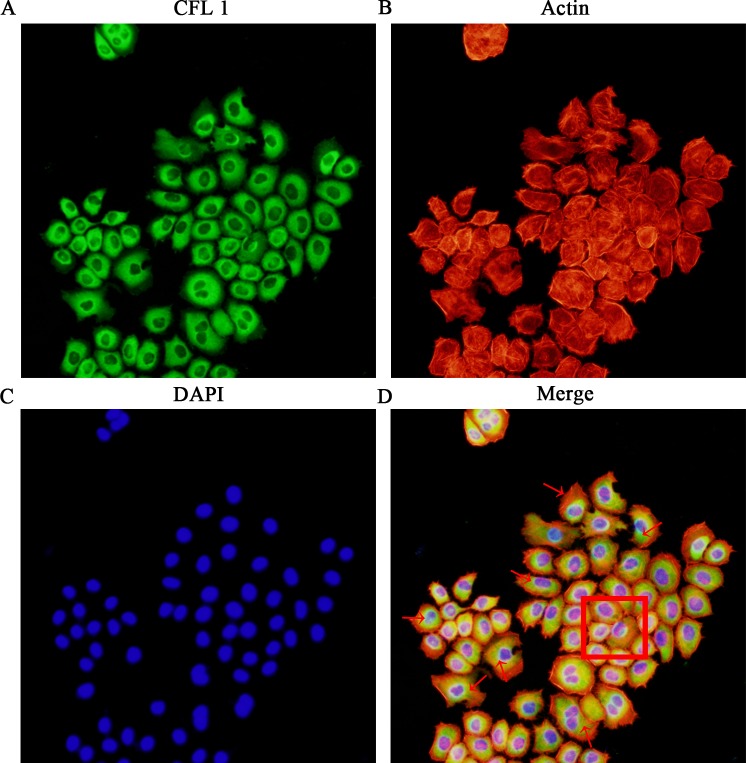
The correlation between CFL1 expression and the EMT **(A)** CFL1 expression in EMT cells. **(B)** Cytoskeletal staining. **(C)** Nuclear DAPI staining. **(D)** CFL1 expression increased as EMT cell lamellipodia and filopodia stretched out. As the red arrow indicates, EMT cells highly expressed CFL1. The square indicates CFL1 expression in NG (negative group) cells. CFL1 was directly involved in the process of EMT (200 ×).

To study the correlation between CFL1 expression and the EMT, we performed immunofluorescence and cytoskeletal staining assays simultaneously in the EMT model. The expression of CFL1 increased in accordance with the stretching out of lamellipodia and filopodia around EMT cells. Compared with the control group cells (shown in the box), EMT cells appeared dark green in the immunofluorescence assay, indicating their high expression of CFL1 (Figure 3). Thus, CFL1 was directly involved in the EMT process in GC cells.

### Lv-CFL1-siRNA

**Figure 4:**
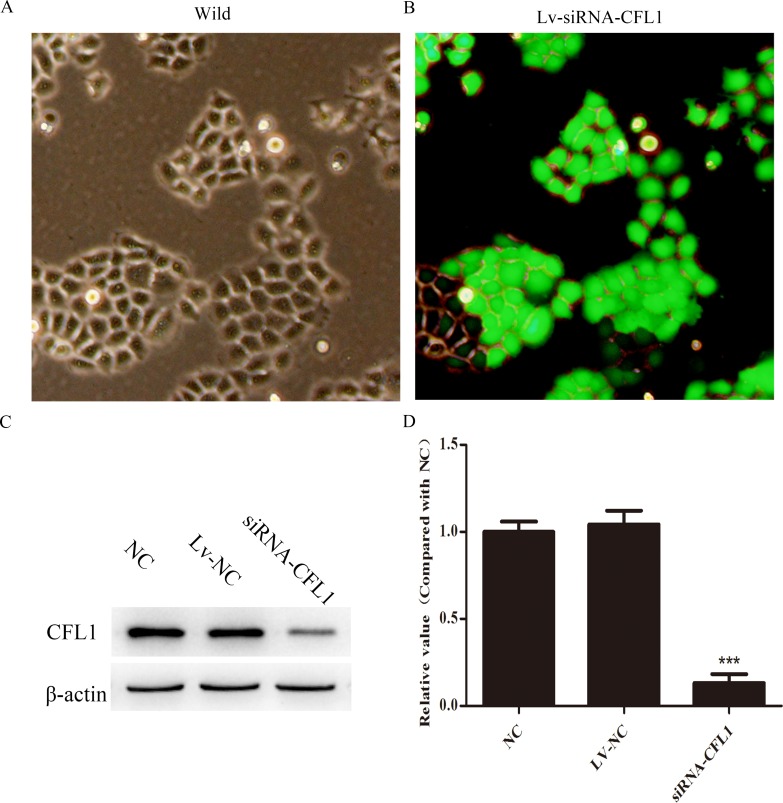
Lv-siRNA-CFL1 silenced CFL1 expression (100×) **(A** and **B)** Expression of green fluorescent protein before and after transfection with lentivirus. **(C** and **D)** Lv-siRNA-CFL1 clearly downregulated CFL1 expression in BGC-823 cells. ***, p < 0.001.

For further studies of the function of CFL1 in GC, lentiviral vectors encoding siRNAs targeting CFL1 (GenBank accession number NM_005507) were constructed by ABM (Nanjing, China). Three different siRNAs targeting CFL1 (Lv-CFL1-siRNA) were designed to ensure the interference effect. The three Lv-CFL1-siRNAs were transfected into BGC-823 cells with Viral-plus Transduction Enhancer G698 and polybrene so that their specificity for CFL1 disruption could be determined. The validated Lv-CFL1-siRNA (Target Seq :AGCATGAATTGCAAGCAAA) was selected for the construction of the lentiviral vector (Figure 4A and 4B). A non-silencing sequence was used as a negative control (Lv-NC). Western blotting revealed that CFL1 was markedly silenced by the Lv-CFL1-siRNA (Figure 4C and 4D).

### Cytoskeletal reorganization and structural basis of the EMT

**Figure 5:**
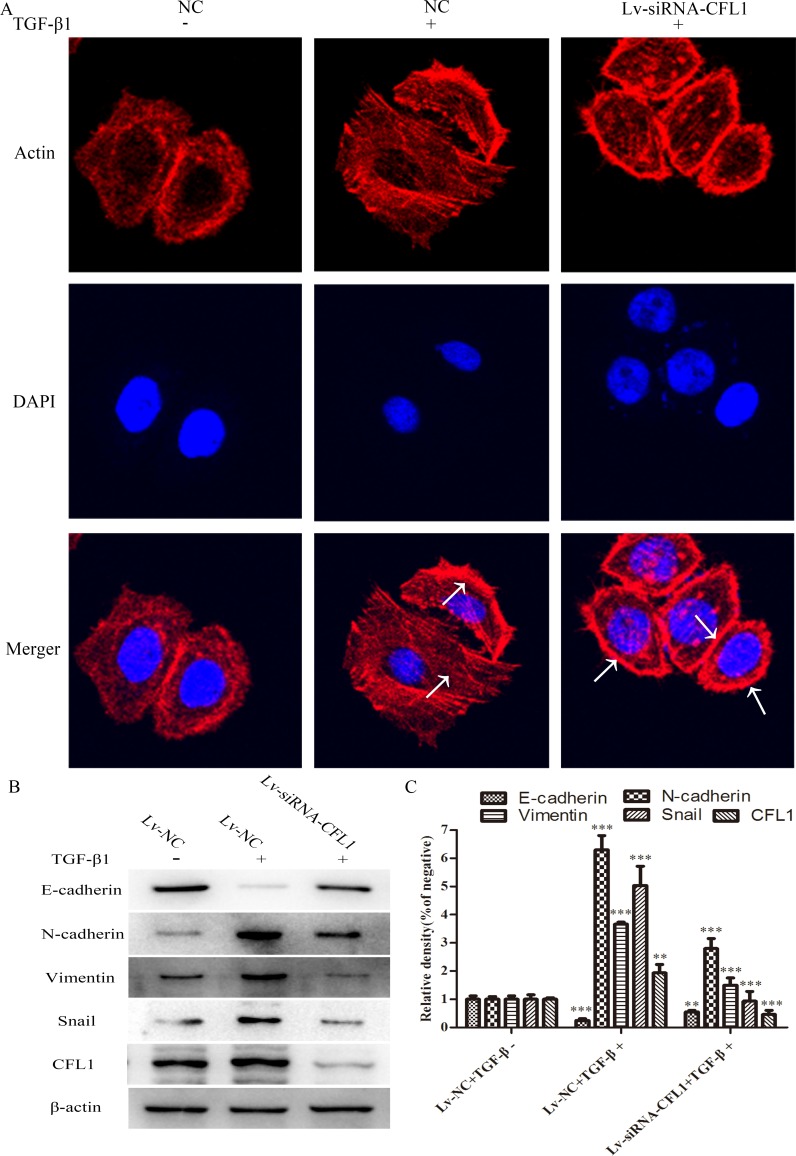
Lv-siRNA-CFL1 suppressed cytoskeletal reorganization and the EMT **(A)** TGF-β1 promoted the activity of actin in the cell. As indicated by the arrow, the microfilaments were evenly distributed in the NC/TGF-β1 group. In the Lv-siRNA-CFL1 group, actin distribution became uneven, and actin was enriched from the cytoplasm to the cell membrane (400×). **(B and C)** EMT-related protein expression was significantly lower in the Lv-siRNA-CFL1 group than in the NC/TGF-β1 group, ** p<0.01, ***p<0.001.

BGC-823 cells were used for a cytoskeletal staining assay in which Lv-siRNA-CFL1 cells were compared with NC cells. After the cells were cultured in the same inducing conditions for 24 hours, it was obvious that Lv-siRNA-CFL1 transfection had inhibited the EMT in terms of both morphology and biomarker protein expression. In addition, the F-actin distribution was uneven and F-actin was enriched from the cytoplasm to the cell membrane when the CFL1 gene was silenced in BGC-823 cells (Figure 5A). EMT-related protein expression was also significantly lower in the Lv-siRNA-CFL1 group than in the NC/TGF-β1 group (Figure 5B and 5C).

### CFL1 induces the EMT by promoting cytoskeletal reorganization

**Figure 6:**
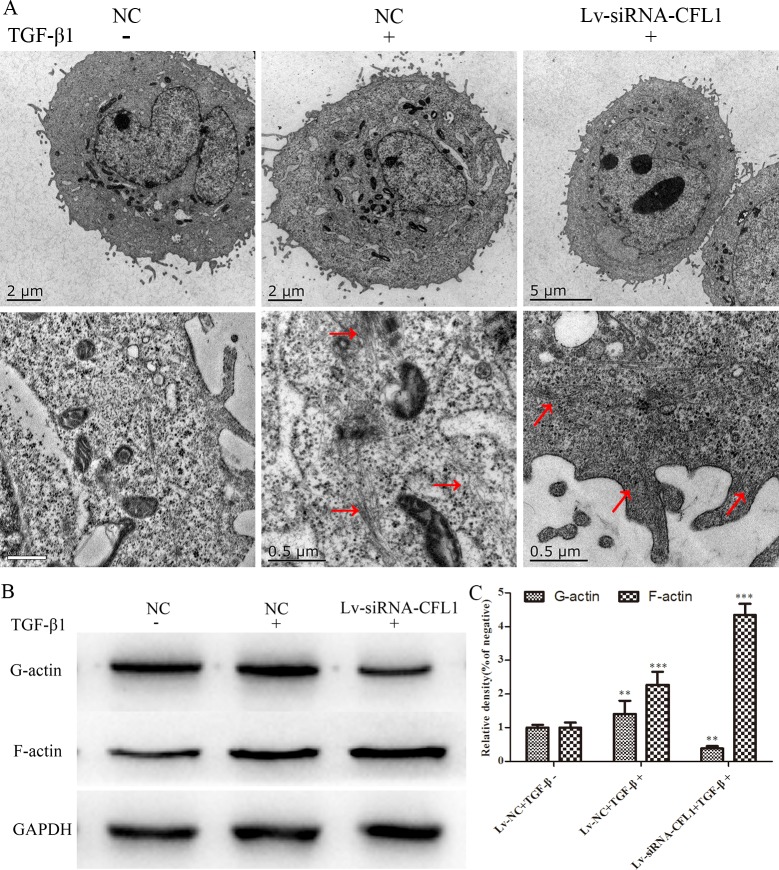
TEM revealed changes in the cell microstructure **(A)** TGF-β1 increased the number of microfilaments in both the cytoplasm and the cell membrane. Lv-siRNA-CFL1 promoted actin aggregation on the cell membrane and the increase of F-actin, but reduced G-actin levels. Similar results were obtained by Western blotting **(B** and **C)**.

As a further verification of the effects of CFL1 on the cytoskeleton, high-resolution TEM was used to observe the changes in the cell microstructure. The number of microfilaments in both the cytoplasm and the cell membrane were greater in the TGF-β1-treated group than in the NC group. Under the same inducing conditions, the number of microfilaments in the Lv-siRNA-CFL1 group also increased, but most of them were concentrated in the cell membrane, and F-actin was significantly increased through G-actin polymerization into F-actin (Figure 6A). Western blotting was also used to detect the levels of F-actin and G-actin. As shown in Figure 6B and 6C, TGF-β1 treatment increased the levels of F-actin and G-actin in the NC group, but only increased F-actin levels in Lv-siRNA-CFL1 group.

### Cell invasion and migration assays

**Figure 7:**
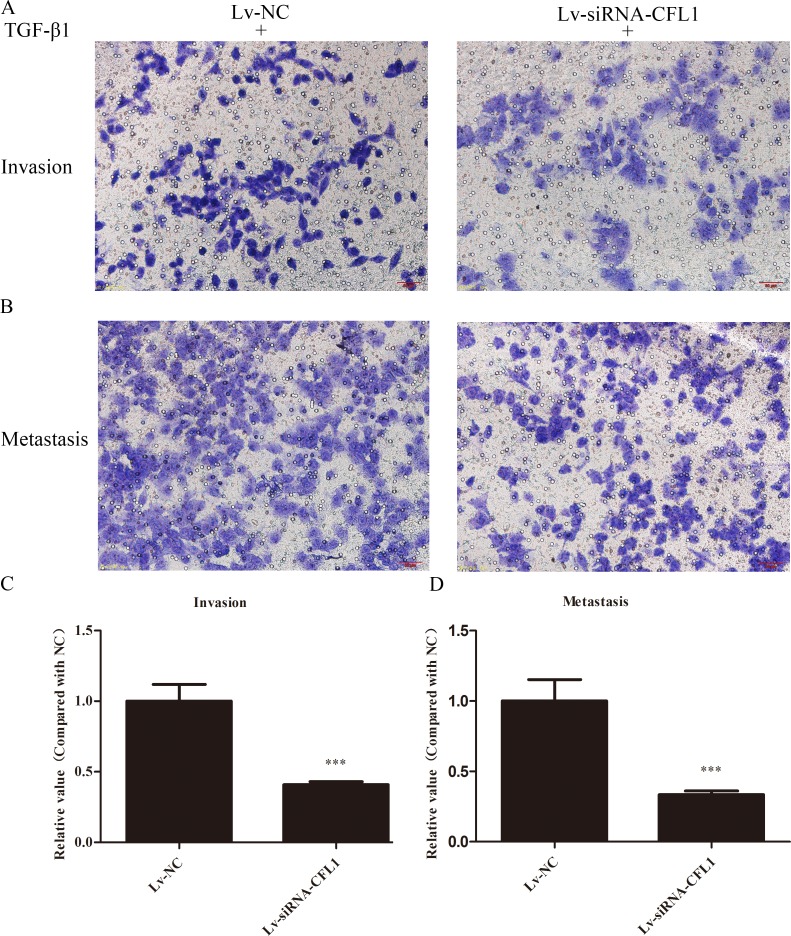
**(A** and **B)** BGC-823 cells achieved high levels of invasion and metastasis after being induced by TGF-β1. The number of cells in Lv-siRNA-CFL1 group penetrating the membrane was significantly reduced in comparison to the Lv-NC group (100×). **(C** and **D)** The penetration of the membrane was clearly inhibited in the Lv-siRNA-CFL1 group relative to the Lv-NC group. ***, p < 0.001.

The EMT is the initial step in the ultimate enhancement of tumor cell invasion and metastasis. We used BGC-823 cells to determine the effects of CFL1 on EMT cell invasion and metastasis. After being cultured in inducing conditions for 24 hours, Lv-NC cells were compared with Lv-siRNA-CFL1 cells. While 10 ng/mL TGF-β1 successfully induced tumor cell EMT, silencing of CFL1 significantly inhibited cell invasion and metastasis (Figure 7).

### Silencing of CFL1 suppresses tumor metastasis *in vivo*

**Figure 8:**
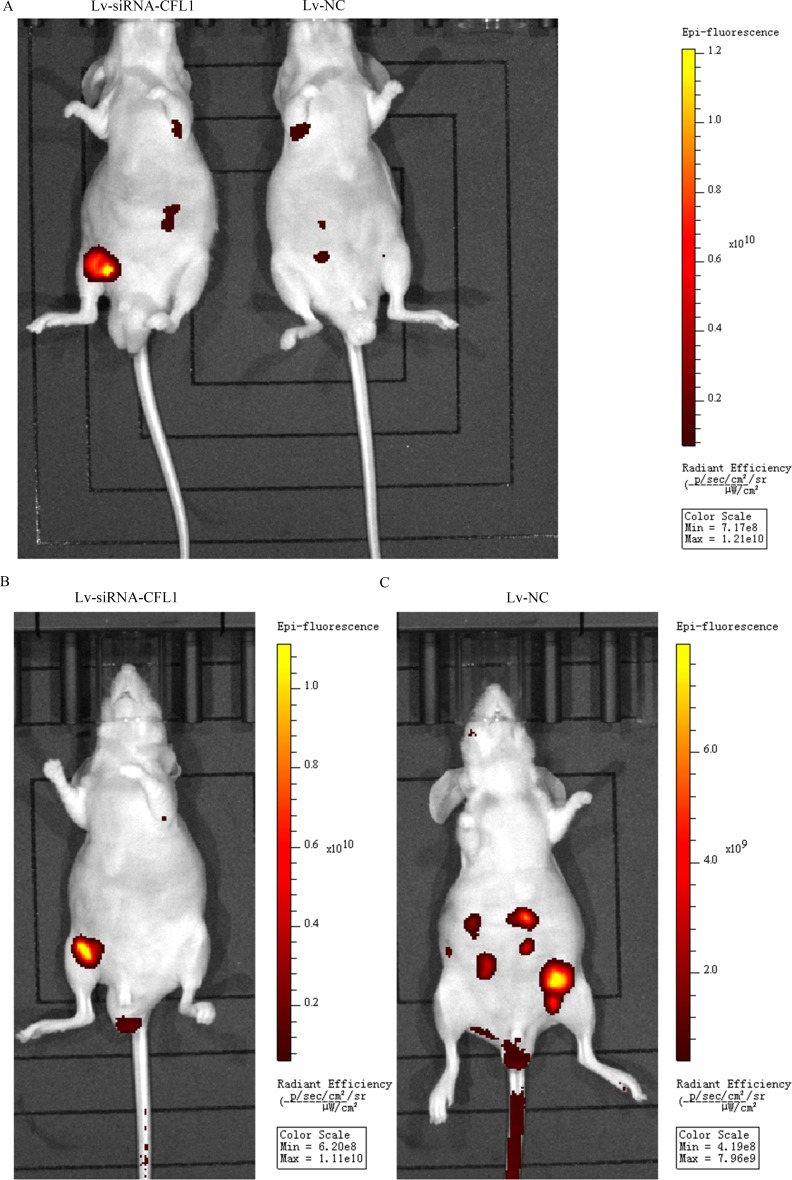
**(A)** Due to tumor metastasis and growth dispersion, the fluorescent signal was weaker in the Lv-NC group than in the Lv-siRNA-CFL1 group (under the same exposure intensity). **(B)** The tumors in the Lv-siRNA-CFL1 group were more concentrated than those in the Lv-NC group. **(C)** The tumors in the Lv-NC group were more dispersed, and there were many tumor metastases.

A nude mouse xenograft model was used to further study the effects of CFL1 inhibition on the EMT *in vivo*. BGC-823 cells transfected with Lv-siRNA-CFL1 or Lv-NC were intraperitoneally injected into nude mice, and tumor metastasis was observed every seven days with a small-animal living imaging system (Figure 8A). The extent of peritoneal carcinomatosis was significantly lower in the Lv-siRNA-CFL1 group than in the Lv-NC group (Figure 8B and 8C). At the same time, the tumor size was measured by the reflected fluorescence signal.

### Metastatic tumors on the mesentery

**Figure 9:**
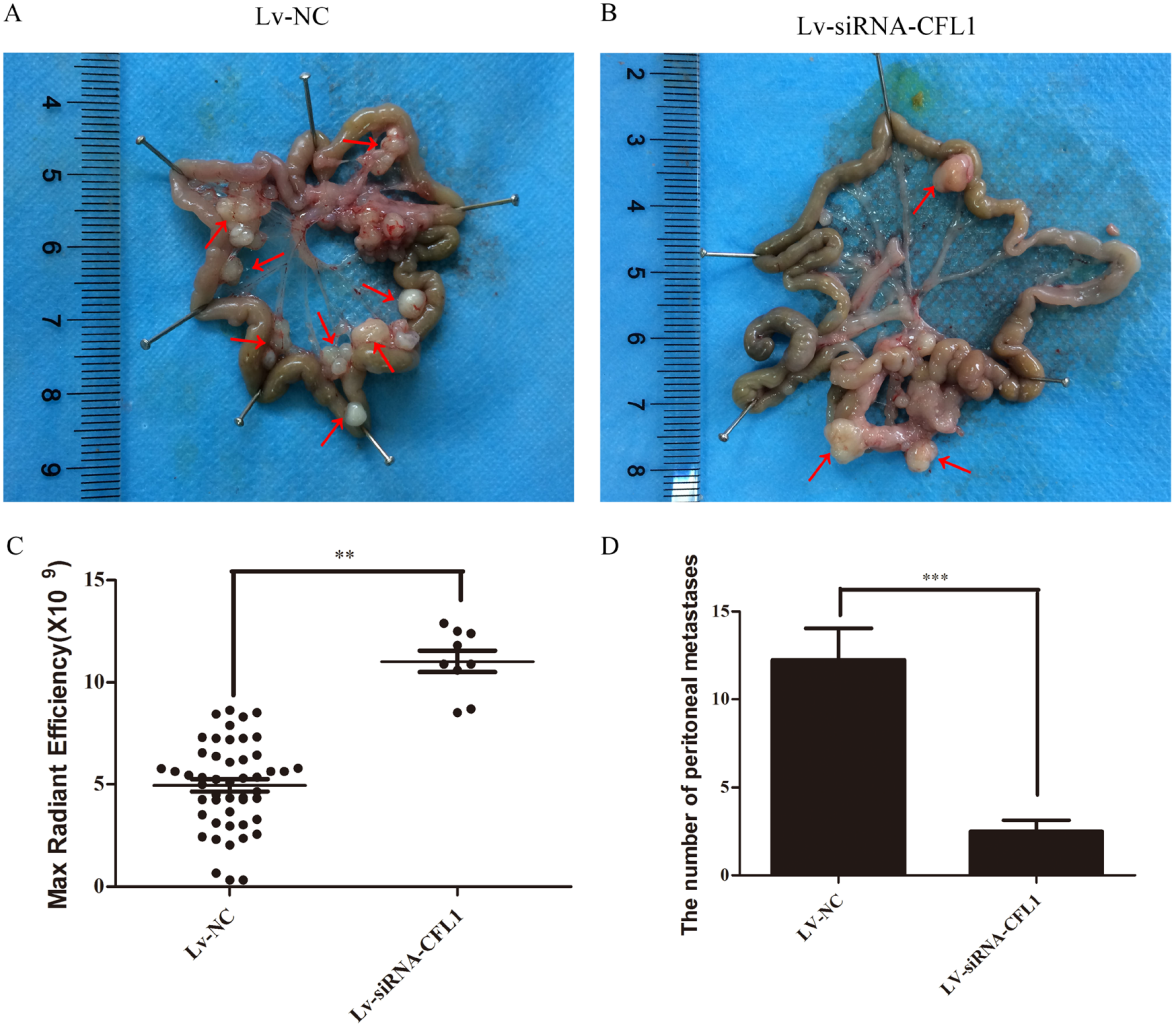
**(A)** Metastatic tumor cells in the peritoneal cavities of Lv-NC nude mice. **(B)** Metastatic tumor cells in the peritoneal cavities of Lv-siRNA-CFL1 nude mice. **(C)** Fluorescence intensity of each metastatic tumor in the Lv-siRNA-CFL1 group and the Lv-NC group. **(D)** The average number of peritoneal metastases in each nude mouse.

To observe the extent of tumor metastasis in the abdominal cavities of the nude mice, we removed and analyzed the mesentery. The results were consistent with the above results from the small-animal living imaging system: peritoneal carcinomatosis was significantly inhibited in the Lv-siRNA-CFL1 group compared to the Lv-NC group (Figure 9A and 9B). The fluorescence signal data and the number of metastases are shown in Figure 9C and 9D.

## DISCUSSION

GC is one of the most common malignant tumors in China, especially in rural areas [1]. Despite the benefits of early diagnosis and standardized surgical treatment, the overall survival of GC patients remains unsatisfactory [17]. Invasion and metastasis severely restrict the survival of GC patients [18–21]. Malignant tumor cells acquire the ability to migrate and invade as a result of the EMT [22]. In this study, we found that silencing of CFL1 significantly inhibited the EMT [15]. In addition, CFL1 expression correlated with cytoskeletal reorganization and EMT biomarker expression in GC. Based on the analysis of 33 clinical specimens, we determined that CFL1 was consistently overexpressed in GC tissues. Therefore, we speculate that CFL1 may promote tumor development.

To further explore the involvement of CFL1 in the malignant behavior of GC, we employed the highly invasive GC cell line BGC-823. We found that CFL1 was overexpressed in the GC cell EMT model induced by TGF-β1. This suggested that CFL1 was involved in the process of EMT, consistent with the data from the clinical samples.

Additional evidence for the function of CFL1 in the EMT was obtained from immunofluorescence combined with a cytoskeletal staining assay. The EMT process under inducing conditions was significantly inhibited after CFL1 was silenced by Lv-siRNA-CFL1. The absence of CFL1 was found to block actin depolymerization and inhibit the repeated disassembly and assembly of the microfilament. G-actin can only be aggregated into F-actin, and F-actin cannot be disaggregated into G-actin. This hindered the enrichment of actin and the formation of pseudopodia on the cell surface. Microscopic evidence provided by high-resolution TEM was used to support the conclusions above. TGF-β1 treatment increased the expression of CFL1, G-actin and F-actin simultaneously. Actin filaments clustered on the cell membrane and cellular microfilament polymerization occurred. Based on the above experimental results, we believe that the silencing of CFL1 can directly inhibit cytoskeletal reorganization and destroy the structural basis for the EMT.

*In vitro* and *in vivo* experiments provided additional support for the important function of CFL1 in the EMT. The silencing of CFL1 significantly inhibited the invasion and metastasis of GC cells *in vitro*. The same results were obtained following *in vivo* transplantation of nude mice.

In summary, we demonstrated that CFL1 induces the EMT by promoting cytoskeletal rearrangement, while silencing of CFL1 inhibits the EMT, invasion and metastasis of GC cells. CFL1 thus appears to be a promising new target for the prognosis and treatment of GC patients.

## MATERIALS AND METHODS

### Patients and tissue specimens

Tumor specimens and non-tumor tissues were obtained from patients who underwent surgical treatment at the Affiliated Hospital of Yangzhou University (Yangzhou, China). None of them had a history of chemotherapy or radiotherapy before sampling, and the diagnosis of GC was pathologically confirmed. This study was approved by the institutional ethics committee of the Affiliated Hospital of Yangzhou University. All patients were asked to sign informed consent forms.

### Reagents and antibodies

RPMI-1640 and fetal bovine serum (FBS) were acquired from Gibco-BRL (MD, USA). Matrigel was purchased from BD Biosciences (New Jersey, USA). TRITC-conjugated Phalloidin was purchased from Sigma Chemical Co (Los Angeles, USA). Recombinant TGF-β1 was obtained from R&D (MN, USA). The fibrous-actin (F-actin) antibody was purchased from NOVUS (Colorado, USA). The globular actin (G-actin) antibody was acquired from Merck (New Jersey, USA). Antibodies against E-cadherin, N-cadherin, Vimentin, Snail, CFL1 and β-actin were purchased from Cell Signaling Technology (MA, USA). Other chemicals of analytical grade were obtained from commercial sources.

### Cell culture and transfection

The human GC cell line BGC-823 was acquired from the Cell Bank of the Chinese Academy of Sciences Shanghai Institute of Cell Biology (Shanghai, China). BGC-823 cells were cultured in RPMI-1640 containing 10% FBS, and were maintained at 37°C in a humidified incubator in an atmosphere of 5% CO_2_. BGC-823 cells (1.5×10^5^) were seeded in six-well plates, or 0.5×10^5^ cells were seeded in 24-well plates and incubated for 12h, then transfected with a lentiviral vector encoding small interfering RNA targeting CFL1 (Lv-siRNA-CFL1). Lv-siRNA-CFL1 was synthesized by ABM (Nanjing, China). Viral-plus Transduction Enhancer G698 and polybrene were used for Lv-siRNA-CFL1 transfection.

### EMT model and changes of cell morphology

BGC-823 cells were plated in six-well dishes for 12h, and RPMI 1640 containing 10 μg/L TGF-β1 was subsequently added to each well and allowed to react for 24 hours. The general morphology, growth and distribution of cells were captured under a microscope. Then, total protein was extracted from each group, and Western blotting was used to detect the expression of EMT-associated proteins. A gel imaging analysis system was used to detect the protein bands of EMT biomarkers.

### Western blot analysis

Cells or tissues were lysed with cold lysis buffer supplemented with a protease inhibitor mixture. The total protein concentration was measured by the BCA assay and was equalized with the extraction reagent. Equivalent amounts of extracts were loaded, subjected to 10% SDS-PAGE, transferred electrophoretically onto PVDF membranes, and analyzed by a Western blotting analysis system.

### The correlation between CFL1 expression and the EMT

Cells were passaged and cultured in suitable media until approximately 50-60% confluent. Cultured cells were fixed with 4% paraformaldehyde for 15-20 minutes at room temperature. After being washed twice, the cells were permeabilized with 0.1% Triton X-100 at room temperature. The cells were again washed twice, and blocking solution (5% BSA) was applied for 30 minutes at room temperature. The primary antibody (anti-CFL1) was diluted to a working concentration with blocking solution and incubated with the cells for 12-18 hours. The cells were then washed twice with 1x wash buffer. The secondary antibody and TRITC-conjugated Phalloidin were diluted with 1x PBS just before use, and were incubated with the cells for 30-60 minutes at room temperature.

### Cell invasion and migration assays

Cell invasion and migration assays were performed with a Transwell membrane according to the manufacturer's instructions. In the invasion assay, Matrigel was applied to the upper chamber. Cells were seeded into the upper chamber, medium containing 10% FBS and TGF-β1 was added to the lower chamber for 24 hours as a chemoattractant. At the end of the treatment, the cells on the upper surface were removed with cotton swabs. Cells that invaded across the Matrigel to the lower surface of the membrane were fixed with methanol and stained with crystal violet. Images were obtained under a microscope at 100× magnification, and invading cells were quantified by manual counting. Migration assays were carried out by the same procedure, except that the polycarbonate membrane was not coated with Matrigel. Each experiment was repeated three times.

### Cell microfilament cytoskeletal staining

Cells were seeded into the 6-wells plates. We established a negative control (NC) group (wild type cell), model group (induced by TGF-β), and Lv-siRNA-CFL1 group (cell transfected with Lv-siRNA-CFL1). Cells were passaged and cultured in suitable media until approximately 50-60% confluent. Then, the cells were fixed with 4% paraformaldehyde for 15-20 minutes at room temperature. Cells in suitable media were covered with dilute TRITC-conjugated Phalloidin in 1x PBS and incubated for 30-60 minutes at room temperature. Following this step, cells were incubated with 4′,6-diamidino-2-phenylindole (DAPI) for 1-5 minutes at room temperature for the purpose of nuclear counterstaining, and were subsequently washed three times (5-10 minutes each) with 1x wash buffer. Fluorescent images can be visualized with a laser scanning confocal microscope (Olympus, Tokyo, Japan).

### Transmission electron microscopy (TEM)

BGC-823 cells in the NC group (wild type cell), Lv-NC/TGF-β group (Cells transfected with Lv-NC) and Lv-siRNA-CFL1/TGF-β group (Cells transfected with Lv-siRNA-CFL1) were washed with PBS, collected and fixed in 2.5% glutaraldehyde for 2 hours at 4°C. The specimens were subsequently rinsed with 0.1 M PBS, fixed in 1% osmium tetroxide for 1-2 hours, progressively dehydrated in 50%, 70%, 80%, 90%, and 100% ethanol for 15 min each, processed for Epon™ embedding, and observed under a transmission electron microscope (CM100; Philips, Netherlands).

### Nude mouse xenograft model

Four-week-old nude mice were purchased from the comparative medicine center of Yangzhou University. All animal experiments were performed in accordance with the guidelines of the National Institutes of Health Guide for the Care and Use of Laboratory Animals. Mice were intraperitoneally injected with 1×10^6^ BGC-823/Lv-NC cells or BGC-823/Lv-siRNA-CFL1 cells. Tumor metastasis was measured every week. The fluorescence signals reflecting the tumor sizes of the mice were collected and analyzed by a small-animal living imaging system (PerkinElmer, USA).

### Statistical analysis

Data were analyzed with SPSS 16.0 statistical software. All data are expressed as the mean ± standard deviation (SD), and P values <0.05 or <0.01 were established as statistically significant. Each experiment was repeated at least three times.

## References

[1] Chen W, Zheng R, Baade PD, Zhang S, Zeng H, Bray F, Jemal A, Yu XQ, He J (2015). Cancer Statistics in China. CA CANCER J CLIN.

[2] Siegel R, Naishadham D, Jemal A (2013). Cancer statistics, 2013. CA Cancer J. Clin.

[3] Ferlay J, Shin HR, Bray F, Forman D, Mathers C, Parkin DM (2010). Estimates of worldwide burden of cancer in 2008: GLOBOCAN 2008. Int J Cancer.

[4] Okimoto RA, Breitenbuecher F, Olivas VR, Wu W, Gini B, Hofree M, Asthana S, Hrustanovic G, Flanagan J, Tulpule A, Blakely CM, Haringsma HJ, Simmons AD (2017). Inactivation of Capicua drives cancer metastasis. Nat Genet.

[5] Zheng X, Carstens JL, Kim J, Scheible M, Kaye J, Sugimoto H, Wu CC, LeBleu VS, Kalluri R (2015). Epithelial-to-mesenchymal transition is dispensable for metastasis but induces chemoresistance in pancreatic cancer. Nature.

[6] Rokavec M, Öner MG, Li H, Jackstadt R, Jiang L, Lodygin D, Kaller M, Horst D, Ziegler PK, Schwitalla S, Slotta-Huspenina J, Bader FG, Greten FR, Hermeking H (2015). Corrigendum. IL-6R/STAT3/miR-34a feedback loop promotes EMT-mediated colorectal cancer invasion and metastasis. J Clin Invest.

[7] Qin Y, Tang B, Hu CJ, Xiao YF, Xie R, Yong X, Wu YY, Dong H, Yang SM (2016). An hTERT/ZEB1 complex directly regulates E-cadherin to promote epithelial-to-mesenchymal transition (EMT) in colorectal cancer. Oncotarget.

[8] Renner G, Noulet F, Mercier MC, Choulier L, Etienne-Selloum N, Gies JP, Lehmann M, Lelong-Rebel I, Martin S, Dontenwill M (2016). Expression/activation of α5β1 integrin is linked to the β-catenin signaling pathway to drive migration in glioma cells. Oncotarget.

[9] Wang W, Mouneimne G, Sidani M, Wyckoff J, Chen X, Makris A, Goswami S, Bresnick AR, Condeelis JS (2006). The activity status of cofilin is directly related to invasion, intravasation, and metastasis of mammary tumors. J Cell Biol.

[10] Tahtamouni LH, Shaw AE, Hasan MH, Yasin SR, Bamburg JR (2013). Non-overlapping activities of ADF and cofilin-1 during the migration of metastatic breast tumor cells. BMC Cell Biol.

[11] Han L, Stope MB, de Jesús ML, Oude Weernink PA, Urban M, Wieland T, Rosskopf D, Mizuno K, Jakobs KH, Schmidt M (2007). Direct stimulation of receptor-controlled phospholipase D1 by phospho-cofilin. EMBO J.

[12] Wang WS, Zhong HJ, Xiao DW, Huang X, Liao LD, Xie ZF, Xu XE, Shen ZY, Xu LY, Li EM (2010). The expression of CFL1 and N-WASP in esophageal squamous cell carcinoma and its correlation with clinicopathological features. Dis Esophagus.

[13] Wu Y, Yi T, Zhang Y (2012). Clinicopathological Significance of cofilin-1 in Gsatric Cancer Tissues. Cancer Research on Prevention and Treatment.

[14] Cho HJ, Baek KE, Kim IK, Park SM, Choi YL, Nam IK, Park SH, Im MJ, Yoo JM, Ryu KJ, Oh YT, Hong SC, Kwon OH (2012). Proteomics-based strategy to delineate the molecular mechanisms of RhoGDI2-induced metastasis and drug resistance in gastric cancer. J Proteome Res.

[15] Zhu Y, Liu Y, Qian Y, Dai X, Yang L, Chen J, Guo S, Hisamitsu T (2014). Research on the efficacy of Celastrus Orbiculatus in suppressing TGF-β1-induced epithelial-mesenchymal transition by inhibiting HSP27 and TNF-α-induced NF-κ B/Snail signaling pathway in human gastric adenocarcinoma. BMC Complement Altern Med.

[16] Su B, Su J, Zeng Y, Liu F, Xia H, Ma YH, Zhou ZG, Zhang S, Yang BM, Wu YH, Zeng X, Ai XH, Ling H (2016). Diallyl disulfide suppresses epithelial-mesenchymal transition, invasion and proliferation by downregulation of LIMK1 in gastric cancer. Oncotarget.

[17] Soleyman-Jahi S, Abdirad A, Fallah AA, Ghasemi S, Sadeghi F, Heidari R, Mahmoodzadeh H, Zendehdel K (2017). Prognostic Significance of Preoperative and Postoperative Plasma Levels of Ghrelin in Gastric Cancer: 3-Year Survival Study. Clin Transl Gastroenterol.

[18] Mikami J, Kimura Y, Makari Y, Fujita J, Kishimoto T, Sawada G, Nakahira S, Nakata K, Tsujie M, Ohzato H (2017). Clinical outcomes and prognostic factors for gastric cancer patients with bone metastasis. World J Surg Oncol.

[19] Kim MS, Kim S (2016). Outcome of Gastric Cancer Surgery in Elderly Patients. J Gastric Cancer.

[20] Ishigami H, Yamaguchi H, Yamashita H, Asakage M, Kitayama J (2016). Surgery after intraperitoneal and systemic chemotherapy for gastric cancer with peritoneal metastasis or positive peritoneal cytology findings. Gastric Cancer.

[21] Yang Y, Zhang J, Yan Y, Cai H, Li M, Sun K, Wang J, Liu X, Wang J, Duan X (2017). Low expression of Rap1GAP is associated with epithelial-mesenchymal transition (EMT) and poor prognosis in gastric cancer. Oncotarget.

[22] Wei SC, Fattet L, Tsai JH, Guo Y, Pai VH, Majeski HE, Chen AC, Sah RL, Taylor SS, Engler AJ, Yang J (2015). Matrix stiffness drives epithelial-mesenchymal transition and tumour metastasis through a TWIST1-G3BP2 mechanotransduction pathway. Nat Cell Biol.

